# A Case of Severe Ovarian Hyperstimulation Syndrome Causing Pleural Effusion

**DOI:** 10.7759/cureus.28804

**Published:** 2022-09-05

**Authors:** Misbahuddin Khaja, Sarah Powell, Sameer Kandhi, Petr Stastka, Diaz Saez Yordanka, Diana M Ronderos

**Affiliations:** 1 Internal Medicine and Pulmonary Critical Care, Icahn School of Medicine at Mount Sinai, Bronx Care Health System, Bronx, USA; 2 Internal Medicine, American University of the Caribbean School of Medicine, Sint Maarten, USA; 3 Internal Medicine, Bronx Care Health System, Bronx, USA; 4 Internal Medicine and Pulmonary Critical Care, Bronx Care Health System, Bronx, USA

**Keywords:** thoracocentesis, pleural effusion, in-vitro fertilization, infertility, ovarian hyperstimulation syndrome

## Abstract

Ovarian hyperstimulation syndrome is one of the complications of treating infertility by ovarian stimulation. As a result of the stimulation, there is a shift of serum from the intravascular space to the third space, leading to complications like ascites and pleural effusion. Here we present a case of a 29-year-old female with polycystic ovarian syndrome who was being treated for infertility using ovarian stimulation agents for in-vitro fertilization. After egg retrieval, the patient complained of shortness of breath and was found to have right-sided pleural effusion. Her symptoms were eventually relieved following an ultrasound-guided diagnostic and therapeutic thoracentesis. Upon discharge, a repeat chest radiograph in the pulmonary clinic showed no pleural effusion. In conclusion, although severe complications like pleural effusion from ovarian stimulation are rare, the physician should be able to recognize this phenomenon to prevent any further deterioration of the patient.

## Introduction

Women undergoing ovarian stimulation therapy for procedures such as in vitro fertilization (IVF) or oocyte retrieval, if not adequately treated, may experience an amplified response leading to ovarian hyperstimulation syndrome (OHSS). It is characterized by increased vascular permeability, shifting proteinaceous fluid into third spaces. Its incidence is increased in younger women, particularly those with lower body weights or those with a history of polycystic ovarian syndrome (PCOS) or OHSS. It is also increased with higher doses of exogenous gonadotropins (human menopausal gonadotrophin, hMG) rather than gonadotrophin-releasing hormone (GnRH) analogues, higher or quickly rising estradiol or an increased number of developing follicles or harvested oocytes. The frequency of OHSS decreases when progesterone is used to reinforce the luteal phase of the menstrual cycle. OHSS is self-limited but exhibits a longer timeline, increased severity, and higher incidence if conception occurs [1]. An increased number of developing follicles is a more significant predictor of disease development compared to estradiol concentration [2].

Signs and symptoms of OHSS occur along a spectrum of severity and usually include lower abdominal or pelvic pain, nausea, vomiting, diarrhoea, and distension. Severe disease is characterized by the presence of pain with any one of the following: pathologic weight gain, ascites, hemodynamic instability with hypotension or tachycardia, respiratory distress or tachypnea, progressive oliguria, acute renal failure, hypovolemic shock, ovarian torsion, abdomen compartment syndrome or laboratory abnormalities [3]. Imaging may show third spacing in the abdomen (ascites) or chest (pleural effusion) [4]. Laboratory workup can show increased hormone levels, hematocrit, leukocytosis, and hypoproteinemia [5].

Before the rise of IVF, the incidence of disease with the use of gonadotrophins varied between 8.4 to 23% for mild OHSS, 0.005 to 7% for moderate disease, and 0.008 to 10% for severe disease [6]. The incidence of OHSS with IVF was 2.1% (1.2% early-type and 0.9% late-type). Therefore, OHSS continues to be a limiting sequela in the clinical use of IVF and other artificial reproductive technologies (ARTs) [7].

This is a case report of severe OHSS in a patient following Follistim/Gonal-F, Menopur cetrotide/ganirelix, and leuprolide IVF before transvaginal oocyte retrieval. The patient did not experience conception. Therefore severe disease occurrence should have been rare.

## Case presentation

A 29-year-old female with a past medical history of hypertension was being treated for polycystic ovarian syndrome (PCOS)-associated infertility with in-vitro fertilization (IVF). She reportedly received ovarian stimulation therapy with Follistim/Gonal-F, Menopur cetrotide/ganirelix, and leuprolide during the in-vitro fertilization (IVF) procedure prior to the transvaginal egg retrieval. The emergency services brought the patient to the hospital from an urgent care clinic, where she presented with worsening chest pain, palpitations, dyspnea on exertion, cough, and bloating for six days following the above procedure.

**Figure 1:**
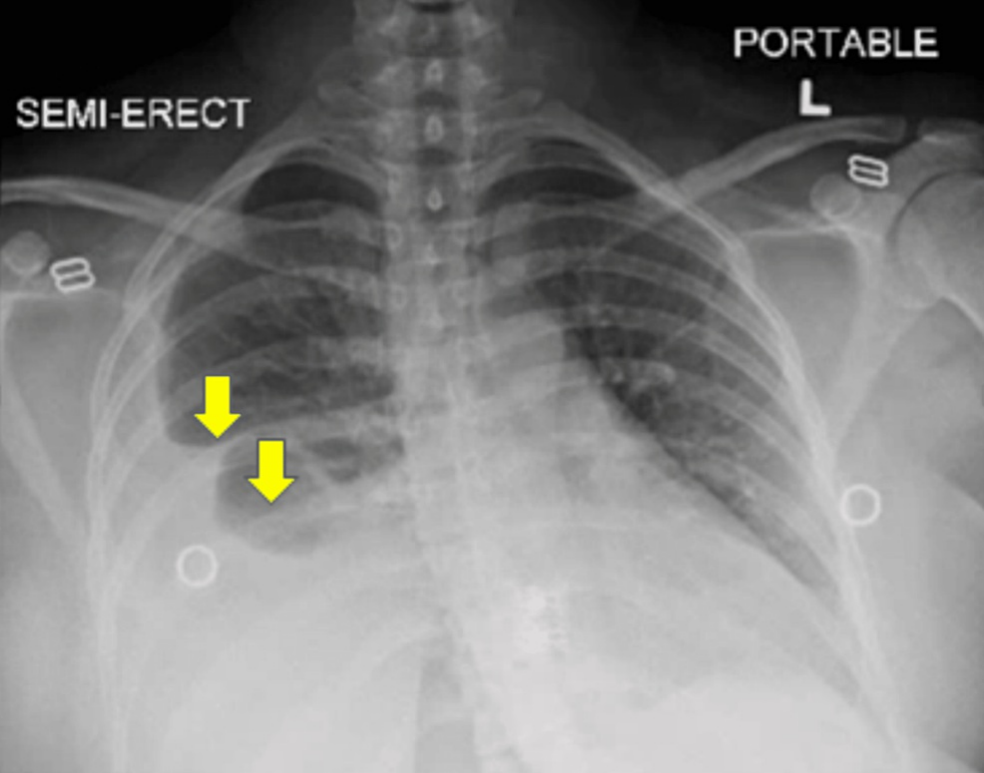
Chest X-ray anterior-posterior view obtained in the emergency department, showing a large right pleural effusion (yellow arrows) and a small left pleural effusion. L: Left side

**Figure 2:**
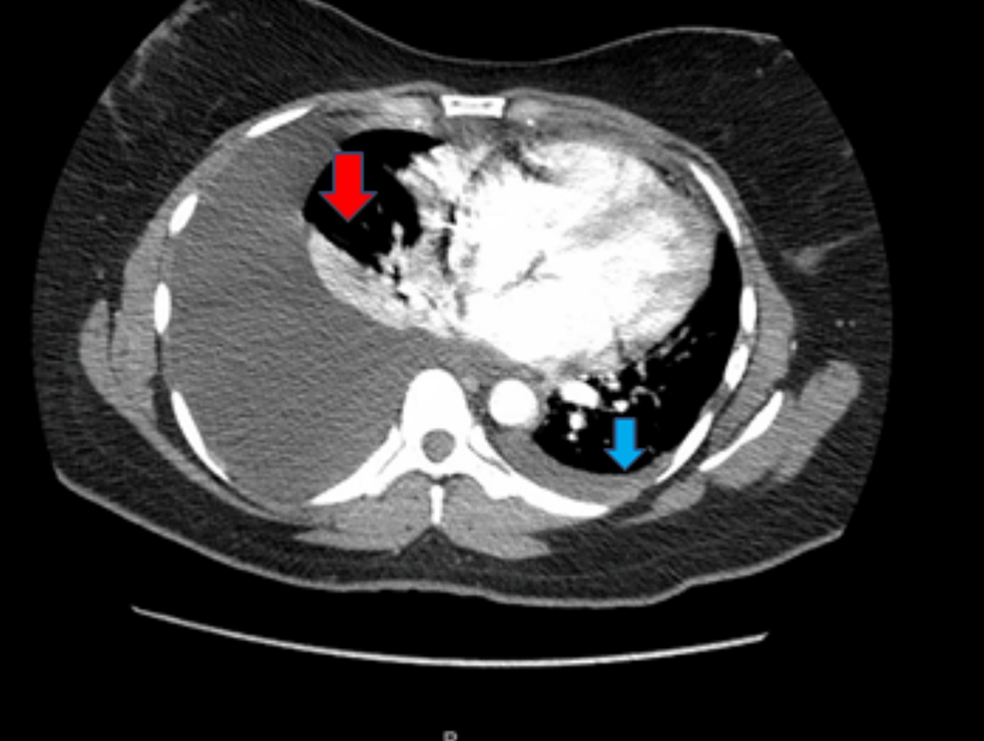
CT chest axial view showing a large right pleural effusion (red arrow) with minimal left pleural effusion (blue arrow).

The patient’s last menstrual period was exactly one month before admission. Initial urine pregnancy testing was negative. Primary vital signs included a blood pressure of 147 mmHg systolic, 96 mmHg diastolic, a pulse of 121 beats per minute, a temperature of 99.5 F, a respiratory rate of 22 bpm, and a SpO2 of 97% on room air. She had a chest computerized tomographic angiogram (CTA) obtained along with initial chest radiographs (CXR). D-dimer levels were elevated on admission, although CTA showed no emboli. CXR (Figure 1) and CTA (Figure 2) revealed a large right pleural effusion and a small left pleural effusion, with associated airspace disease of atelectasis or pneumonia. A leftward mediastinal shift was present, likely secondary to the larger right-sided pleural effusion and ascites. The laboratory results obtained in the emergency department were summarized in Table 1. The serum C-reactive protein (CRP) levels were elevated.

**Table 1:** Laboratory values on admission

Lab Tests	Lab Values	Normal Range
WBC count	8.8 k/uL	4.8-10.8 k/uL
RBC Count	5.31 MIL/uL	4.50-5.90 MIL/uL
HGB	14.9 g/dL	12.0-16.0 g/dL
Hematocrit	44.0 %	42-51 %
Platelet	280 k/uL	150-400 k/uL
General Chemistry		
Sodium, Serum	135 mEq/L	135-145 mEq/L
Potassium, Serum	4.3 mEq/L	3.5-5.0 mEq/L
Blood Urea Nitrogen, Serum	6.0 mg/dL	8-26 mg/dL
Creatinine, Serum	0.6 mg/dL	0.5-1.5 mg/dL
Bilirubin, Serum total	0.5 mg/dL	0.2-1.1 mg/dL
Bilirubin, Serum Direct Conjugated	0.2 mg/dL	0.0-0.3 mg/dL
Alkaline Phosphatase, Serum	54 unit/L	56-155 unit/L
Aspartate Transaminase, Serum	31 unit/L	9-48 unit/L
Alanine Aminotransferase, Serum	21 unit/L	5-40 unit/L
Lactic acid Level	1.5 mmoles/L	0.5-1.6 mmoles/L
C- Reactive Protein	21.16 mg/L	5 mg/L
D-dimer Assay, Plasma	1077 ng/mL	0-230 ng/mL
Lactate Dehydrogenase, Serum	250 unit/L	110-210 unit/L
Thyroid Stimulating Hormone	2.99 mIU/L	0.40-4.50 mIU/L
Urine Toxicology	Negative	
Anti Nuclear Antibody	Positive , 1:40, Speckled	
Anti- DNA Antibody	<1.0	<1.0
Anti Myeloperoxidase Antibody	<1.0	<1.0
Anti Proteinase 3 Antibody	<1.0	<1.0
Anti Rho Antibody	<1.0	<1.0
Anti La Antibody	<1.0	<1.0
C4 Complement, Serum	16 mg/dl	16-47 mg/dl
Rheumatoid Factor, Serum	<10 mg/dl	14 mg/dl

Physical examination while in the medical unit showed hirsutism, obesity, decreased breath sounds in the right lung, decreased tactile fremitus, and dullness to percussion. In addition, the patient’s heart rate at this time had increased to 133 bpm. The patient’s PCOS was managed here with metformin 500mg QD, along with monitoring of hemoglobin A1C and point-of-care glucose levels. Her hypertension was managed with her home dose of amlodipine 5mg QD.

Ultrasound-guided thoracocentesis was performed for the patient’s right-sided pleural effusion on the day of admission. The procedure yielded 1000ml of hazy, dark, straw-colored fluid. Laboratory analysis of the pleural fluid is summarized in Table 2. A pleural drainage catheter was left in place for continued drainage. Post-procedure CXR showed decreased size of the pleural effusion with mildly improved aeration of the right lung. An obstetrics and gynecology (OBGYN) consult was also obtained on admission. Transvaginal ultrasound (TVUS) and abdominal ultrasound were obtained to assess for ascites at their request.

**Table 2:** Pleural fluid analysis WBC: white blood cell; RBC: red blood cell; PCR: polymerase chain reaction

Pleural Fluid analysis	Lab Values	Normal Values
Color, Fluid	Brown	
Appearance	Hazy	
pH, Fluid	7.0	
WBC Count, Fluid	82 cells/cu.mm	
RBC Count, Fluid	11,950 million cells/cu.mm	
Segmented Neutrophils, Fluid	46%	
Lymphocyte Count, Fluid	54%	
Microscopy, Fluid	Pyknotic nuclei were present, and many mesothelial cells along with macrophages were seen in the fluid.	
Glucose, Fluid	81 mg/dl	50-80 mg/dl
Albumin, Fluid	3.4 mg/dl	
Protein, Fluid	4.5 g/dl	
Lactate Dehydrogenase, Fluid	215 units/L	100-190 units/L
Triglycerides, Fluid	19 mg/dl	
Amylase, Fluid	26 U/L	30-110 U/L
Cholesterol, Fluid	34.9 mg/dl	
Adenosine Deaminase, Fluid	8.9 U/L	<9.2 U/L
Culture, Fluid	Negative	
PCR for Mycoplasma Tuberculosis, Fluid	Negative	

**Figure 3:**
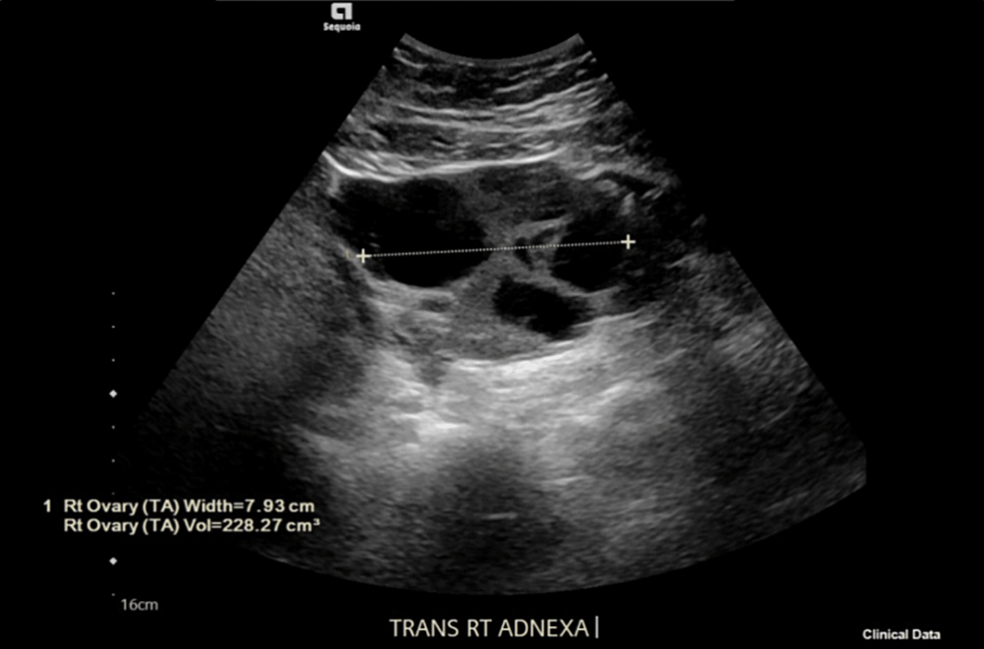
TVUS transverse right adnexa, total 7.93 cm in width, with a 3.5cm diameter simple cyst TVUS: transvaginal ultrasound; TV: transvaginal; TRANS RT ADNEXA: transverse right adnexa

**Figure 4:**
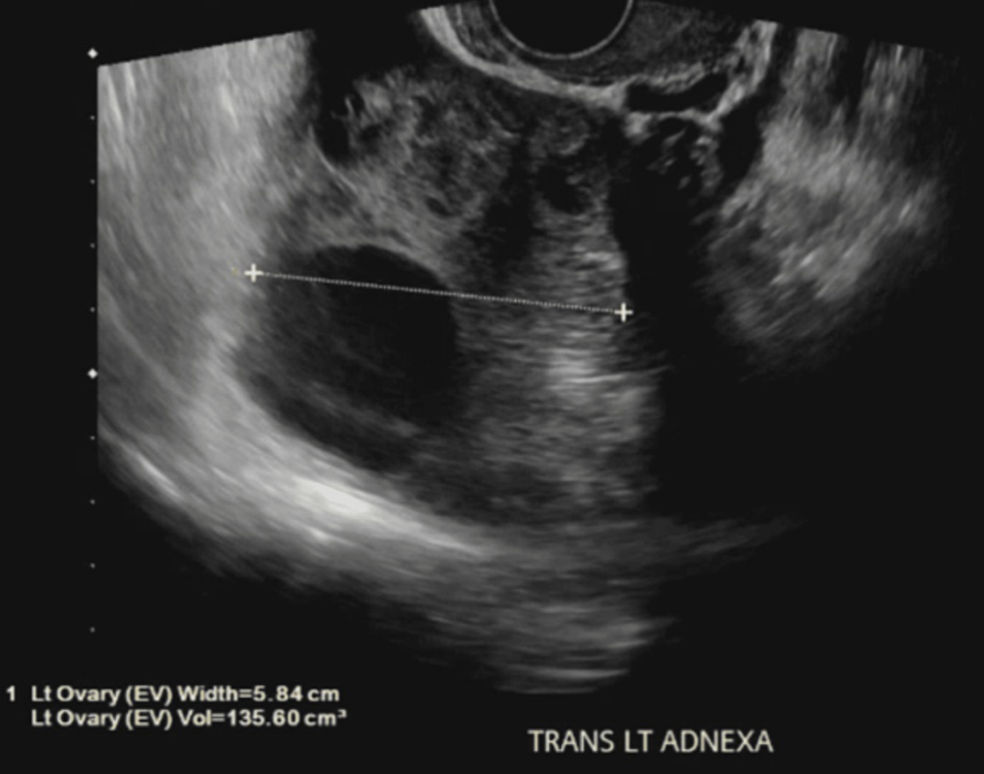
TVUS transverse left adnexa, 5.84 cm in width, with a simple cyst. TVUS: transvaginal ultrasound; EV: endovaginal; TRANS LT ADNEXA: transverse left adnexa

One day post-procedure, the patient reported shortness of breath, although repeat CXR showed near-complete resolution of the right-sided pleural effusion. A 12-lead electrocardiogram showed normal sinus rhythm. Linear atelectasis in the right middle lobe and bilaterally interstitial prominence of the lower lobes was also noted. Venous ultrasound of the lower extremities was negative for deep venous thrombosis. A pulmonary consult was obtained, with no new or further recommendations added. Pelvic ultrasound findings were consistent with ovarian hyperstimulation syndrome. The right ovary contained multiple enlarged follicles and a simple cyst 3.5cm in diameter (Figure 3). Similarly, the left ovary had multiple enlarged follicles and a simple cyst measuring 4.3cm in diameter (Figure 4). 

A minimal amount of free fluid was present in the pelvic cul-de-sac near the uterine fundus. TVUS confirmed these findings. Abdominal ultrasound proved the absence of ascites in all quadrants. Sputum mycobacterium cultures showed no growth. Initial rheumatology workup showed negative DNA antibodies, myeloperoxidase and proteinase-3 antibodies, cryptococcal antigen, cytology, and HIV 1/2/O antibodies.

On Day 4 of admission, effusion drainage was attempted for a final time, with 30cc of drainage, so the pigtail was removed. Bedside chest ultrasound showed lung sliding and seashore signs, confirming the absence of pneumothorax. Repeat CXR post-procedure confirmed the absence of effusion and pneumothorax. A rheumatology consult was requested due to a mildly positive ANA titer of 1:40 with a nuclear, speckled pattern. Rheumatology recommended obtaining anti-Smith antibody, C3/C4, lupus anticoagulant, and antiphospholipid antibody titers, which were within normal limits.

**Figure 5:**
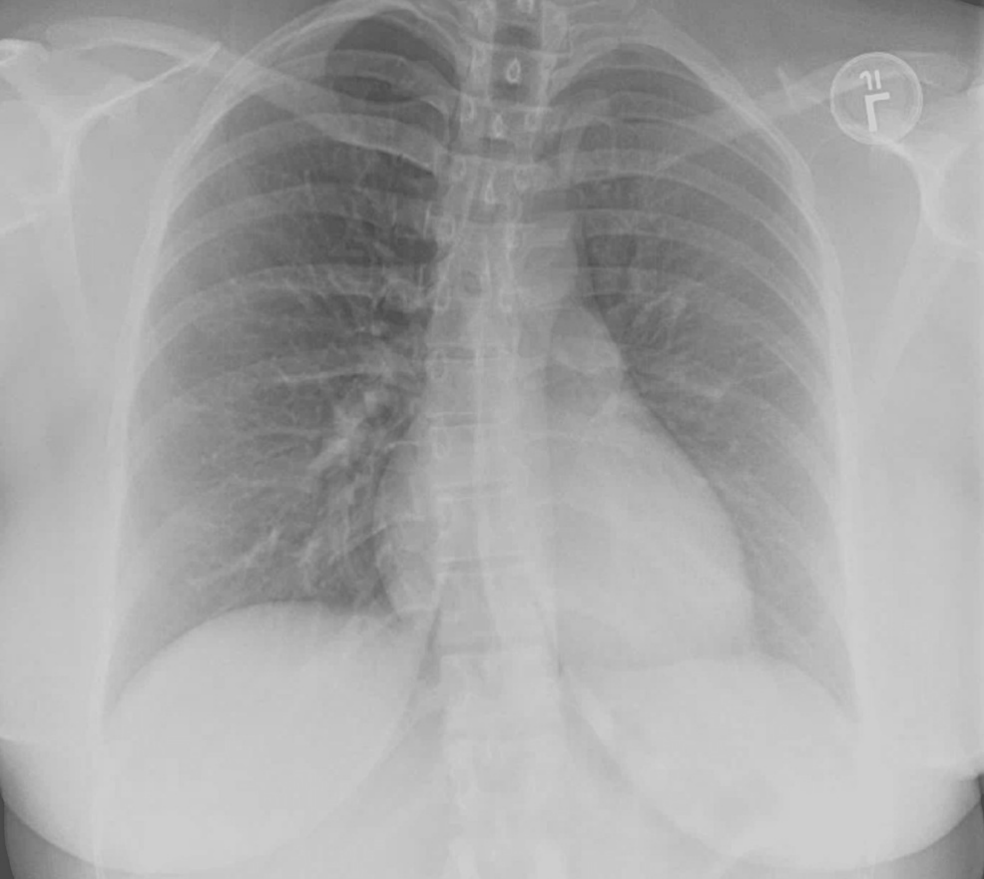
Chest X-ray posterior-anterior view obtained in the pulmonary clinic on follow up, showing resolution of the right and left pleural effusion.

The patient was discharged home with three additional days of cefpodoxime 200mg bid. A follow-up appointment was also scheduled with a pulmonary specialist at a later date. Additional lab results obtained at rheumatology follow-up included negative RPR and FTA, DRVVT, complete antiphospholipid panel, anti-Smith antibody, anti-RNP antibody, SS-A, and SS-B antibodies, and antibody to antiscleroderma-70. Serum C3 was 164.0mg/dL (N=90-150). The chest radiograph at the pulmonary clinic showed no re-accumulation of pleural fluid (Figure 5).

## Discussion

Ovarian hyperstimulation syndrome (OHSS) can be precipitated by procedures such as in-vitro fertilization (IVF) or oocyte retrieval [1]. The incidence of OHSS before modern assisted reproductive technology (ART) was significantly more significant, at least when considering mild disease [6]. Comparatively, incidences of moderate and severe OHSS resulting from older vs. newer ARTs have been more difficult to conclude [2]. Presently, the understood frequency of incidence for moderate to severe OHSS is approximately 1-5% in all IVF cycles, with an associated mortality of 1 in 45,000-50,000 individuals undergoing the procedure [8]. In the last two decades, there has been an increase in new cases of OHSS, likely due to the rising popularity of ART worldwide. Though the newer ART protocols in the previous decade have significantly shifted from utilizing human chorionic gonadotropin(hCG) and human menopausal gonadotropin (hMG) preparations to using gonadotropin-releasing hormone agonists, resulting in decreased incidence of OHSS, it has not been successful in completely eliminating it [9].

OHSS presents more commonly in younger females with lower BMIs, especially those with PCOS, as in this patient [1]. OHSS presents clinically with nausea, vomiting, and abdominal discomfort, likely linked to ovarian enlargement and ascites. OHSS is further linked to an increase in vascular permeability, regulated by the renin-angiotensin-aldosterone system (RAAS) and also with the release of vascular endothelial growth factor (VEGF) and interleukins (ILs), which modulate the permeability of vascular beds [8].

This case presents a patient with a severe presentation of OHSS, following ovarian stimulation therapy during the IVF procedure prior to transvaginal egg retrieval. The OHSS is marked by a large right pleural effusion and a small left pleural effusion, with associated airspace disease of atelectasis or pneumonia. In addition, a corresponding leftward mediastinal shift was present, along with ascites. According to a systematic review of 30 cases of OHSS in 2018 by Irani et al., 86.6% of patients presented clinically with dyspnea, and 80% had pleural effusions on the right side. In addition, 90% of these patients underwent thoracocentesis, which yielded ⅔ exudate and ⅓ transudate [10].

The pathophysiology of OHSS is not fully understood. However, a leading hypothesis of the pathogenesis of the fluid accumulation delineates that fluid flows from the peritoneal cavity into the pleural space, caused by the pressure differential across the thoracic duct and diaphragmatic defects - which are more commonly found on the right side. This process is likely mediated by the release of vasoactive substances like VEGF, IL-1, IL-2, and IL-6, which facilitate vascular permeability. Thereby, right-sided pleural effusions appear more likely in the presence of OHSS [8].

OHSS is still a rare complication of IVF, and there are only fewer guidelines [11] presently put in place for recognizing and acting on this finding. Early detection and prevention methods may be crucial in identifying and treating OHSS. Our patient presented to the ED with early signs, including dyspnea, and was promptly treated, likely contributing to a positive hospital course. There is no consensus on an acceptable classification system for OHSS. However, the Royal College of Obstetricians and Gynaecologists proposed a symptom and severity-based classification system in 2016, which may be helpful as a reference when attempting to categorize and diagnose this finding. According to these guidelines, this case would likely be classified as early onset stage III, severe. This grade correlates with the conclusions in this case, including ascites, pleural effusion, and hypoproteinemia. However, it doesn’t include other suggested findings, including electrolyte depletion, hypovolemia, or ovarian size >12cm each. These criteria should be used as one possible guide for physicians seeking to grade the severity of OHSS, but not as a complete ruleset [8].

The first step in preventing OHSS is for clinicians to inform themselves of its possibility when dealing with ARTs. While there is currently no available intervention for preventing OHSS, a 2016 pharmacologic systematic review and network meta-analysis found several candidate drugs for possible prevention and management of OHSS [7]. According to the results, aspirin, IV calcium, cabergoline, IV hydroxylethyl starch (HES), and metformin have all demonstrated preventive potential in cases of OHSS, with no harmful effect on pregnancy rate [2]. Notably, aspirin and IV calcium demonstrated the highest effectiveness. Furthermore, the study showed that metformin should be used as prophylaxis in patients with PCOS without a poor ovarian reserve [7].

## Conclusions

With the anticipated sustained rise of the use of in vitro fertilization and other assisted reproductive technologies, ovarian hyperstimulation syndrome must be a topic of continued research. The development of a systematic guideline for diagnosis and treatment is vital to the well-being of patients. Furthermore, physician education should focus on its early detection to avoid more severe manifestations. 

## References

[1] Practice Committee of American Society for Reproductive Medicine (2008). Ovarian hyperstimulation syndrome. Fertil Steril.

[2] Papanikolaou EG, Pozzobon C, Kolibianakis EM (2006). Incidence and prediction of ovarian hyperstimulation syndrome in women undergoing gonadotropin-releasing hormone antagonist in vitro fertilization cycles. Fertil Steril.

[3] Timmons D, Montrief T, Koyfman A, Long B (2019). Ovarian hyperstimulation syndrome: A review for emergency clinicians. Am J Emerg Med.

[4] Kumar P, Sait SF, Sharma A, Kumar M (2011). Ovarian hyperstimulation syndrome. J Hum Reprod Sci.

[5] Navot D, Bergh PA, Laufer N (1992). Ovarian hyperstimulation syndrome in novel reproductive technologies: prevention and treatment. Fertil Steril.

[6] Schenker JG, Weinstein D (1978). Ovarian hyperstimulation syndrome: a current survey. Fertil Steril.

[7] Guo JL, Zhang DD, Zhao Y, Zhang D, Zhang XM, Zhou CQ, Yao SZ (2016). Pharmacologic interventions in preventing ovarian hyperstimulation syndrome: a systematic review and network meta-analysis. Sci Rep.

[8] Vidal A, Wachter C, Kohl Schwartz A, Dhakal C (2021). A rare presentation of isolated right-sided pleural effusion in the context of ovarian hyperstimulation syndrome: A case report. Case Rep Womens Health.

[9] Blumenfeld Z (2018). The Ovarian hyperstimulation syndrome. Vitam Horm.

[10] Irani M, Robles A, Gunnala V, Chung P, Rosenwaks Z (2018). Unilateral pleural effusion as the sole clinical presentation of severe ovarian hyperstimulation syndrome: a systematic review. Gynecol Endocrinol.

[11] The Eshre Guideline Group On Ovarian Stimulation, Bosch E, Broer S (2020). ESHRE guideline: ovarian stimulation for IVF/ICSI. Hum Reprod Open.

